# OLS Dialog: An open-source front end to the Ontology Lookup Service

**DOI:** 10.1186/1471-2105-11-34

**Published:** 2010-01-17

**Authors:** Harald Barsnes, Richard G Côté, Ingvar Eidhammer, Lennart Martens

**Affiliations:** 1 Department of Informatics, University of Bergen, Bergen, Norway; 2 EMBL Outstation, European Bioinformatics Institute (EBI), Wellcome Trust Genome Campus, Hinxton, Cambridge, UK; 3 Department of Medical Protein Research, VIB, B-9000 Ghent, Belgium; 4 Department of Biochemistry, Ghent University, B-9000 Ghent, Belgium

## Abstract

**Background:**

With the growing amount of biomedical data available in public databases it has become increasingly important to annotate data in a consistent way in order to allow easy access to this rich source of information. Annotating the data using controlled vocabulary terms and ontologies makes it much easier to compare and analyze data from different sources. However, finding the correct controlled vocabulary terms can sometimes be a difficult task for the end user annotating these data.

**Results:**

In order to facilitate the location of the correct term in the correct controlled vocabulary or ontology, the Ontology Lookup Service was created. However, using the Ontology Lookup Service as a web service is not always feasible, especially for researchers without bioinformatics support. We have therefore created a Java front end to the Ontology Lookup Service, called the OLS Dialog, which can be plugged into any application requiring the annotation of data using controlled vocabulary terms, making it possible to find and use controlled vocabulary terms without requiring any additional knowledge about web services or ontology formats.

**Conclusions:**

As a user-friendly open source front end to the Ontology Lookup Service, the OLS Dialog makes it straightforward to include controlled vocabulary support in third-party tools, which ultimately makes the data even more valuable to the biomedical community.

## Background

The amount of biomedical data stored in public databases has grown extensively in the last couple of decades and will most likely continue to increase just as rapidly in the coming years [1]. However, for researchers to make optimal use of this large amount of information, it has to be structured and annotated in such a way that data from different labs, different instruments and even of different types can be compared and analyzed efficiently. Data must therefore be annotated using precisely defined terms agreed upon by all data providers. With this requirement in mind, controlled vocabularies (CV) and ontologies have been created. A CV is defined as a limited list of clearly defined terms, with optional relationships between the terms, while an ontology moves beyond a mere CV by attempting to extensively model a part of the real world [2].

But even though systems for annotating biomedical data in consistent ways are available, finding and using the correct CV terms to annotate a data set may in some cases be a difficult task. Partly as a response to this the Ontology Lookup Service (OLS, http://www.ebi.ac.uk/ols) was created [3,4]. The OLS provides interactive and programmatic interfaces to query, browse and navigate a long list of biomedical ontologies, thus making it easier to find the desired CV terms. However, using the OLS as a web service is not always feasible, especially for researchers without bioinformatics support.

We have therefore created a Java front end to the OLS, called the OLS Dialog http://ols-dialog.googlecode.com, which can be plugged into any application requiring the annotation of data using CV terms, making it straightforward to find and use CV terms without requiring any additional knowledge about web services or ontology formats.

## Implementation

The OLS Dialog has been implemented in Java, is platform independent and requires Java 1.5 (or newer). As the name suggests, the OLS Dialog is implemented as a Java dialog, which depends on a parent frame or dialog. Selected terms are communicated to this parent through the OLSInputable interface, defined in the package no.uib.olsdialog. This interface contains two simple methods that fully represent the interaction of the OLS Dialog with its parent.

Platform independent Java binaries, additional documentation and source code are freely available at http://ols-dialog.googlecode.com. OLS Dialog is released under the permissive Apache2 license http://www.apache.org/licenses/LICENSE-2.0.html allowing for easy reuse of the code and tool in other settings.

## Results

Four different CV term search strategies are supported in the OLS Dialog: (i) Term Name Search, locates a CV term by a (partial) match to a search term; (ii) Term ID Search, locates a CV term by its CV term accession number; (iii) PSI-MOD Mass Search, finds the CV term for a modification in the PSI-MOD ontology [5] using the mass of the modification; and (iv) Browse Ontology, browses the ontology as a tree structure and allows the user to locate the desired term. Furthermore, OLS Dialog also provides a Term Hierarchy Graph view that can be used to locate or verify a CV term by inspecting the term hierarchy. Note that the Term Name Search supports both fuzzy/partial searches ('oxid' locates all partially matching CV terms, e.g., 'oxidation' and 'L-cystine S-oxide') and synonym searches (MOD:00045 can be found by searching for 'pros-phosphohistidine', 'phosphorylation', 'Npi-phosphorylated L-histidine', etc).

The main interface of the OLS Dialog is split into three main parts. At the top, the desired ontology is selected. At the time of writing more than 70 different biomedical ontologies are supported in the OLS, including over 900 000 CV terms. A full list of the supported ontologies can be found at http://www.ebi.ac.uk/ols. These ontologies are constantly updated and maintained by specialists in the various fields [6-8], and new changes will be automatically picked up daily by the OLS. It is important to note that the OLS Dialog does not store the ontologies locally but accesses the OLS web service whenever a search is performed. This ensures that the latest versions of the ontologies are always used.

In addition to searching a specific ontology it is also possible to search in all ontologies at once by selecting 'Search In All Ontologies' at the top of the list. This makes it possible to locate a CV term for which the ontology is unknown. Searching in all ontologies slows down the search however, and is not the recommended standard search option.

Below the ontology selection there are four tabs, one for each search option. Although each tab provides a search-specific interface, the overall structure stays the same. The search parameters are inserted or selected at the top of the tab, and the results of the search, i.e., the matching CV terms, are inserted into the 'Search Results' table. By selecting a CV term in the results table the term's associated details will be presented in the 'Term Details' table. The Browse Ontology tab is slightly different, as it replaces the 'Search Results' table with a tree structure of all terms in the currently selected ontology. It is also possible to view the term hierarchy as a graph by clicking the 'View Term Hierarchy' link at the top of the 'Term Details' text area. When a CV term is selected in the table (or in the tree) clicking the 'Use Selected Term' sends the selected term to the parent frame or dialog.

**Figure 1:**
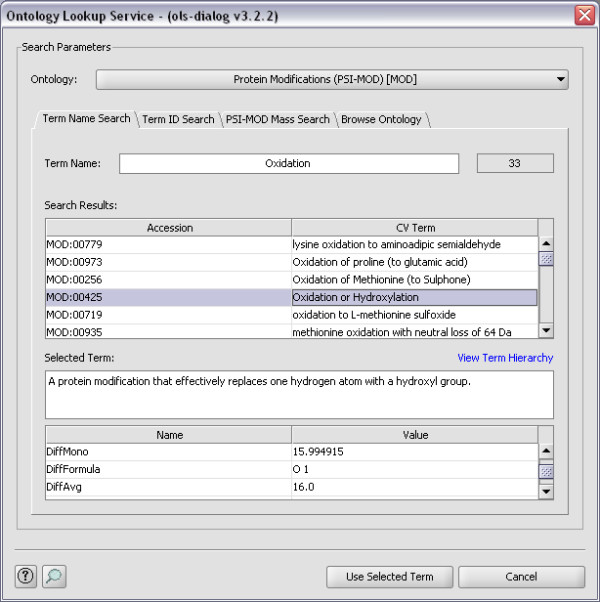
**Term Name Search**. Here the OLS Dialog finds the possible CV terms for the search term 'Oxidation', using Term Name Search.

**Figure 2:**
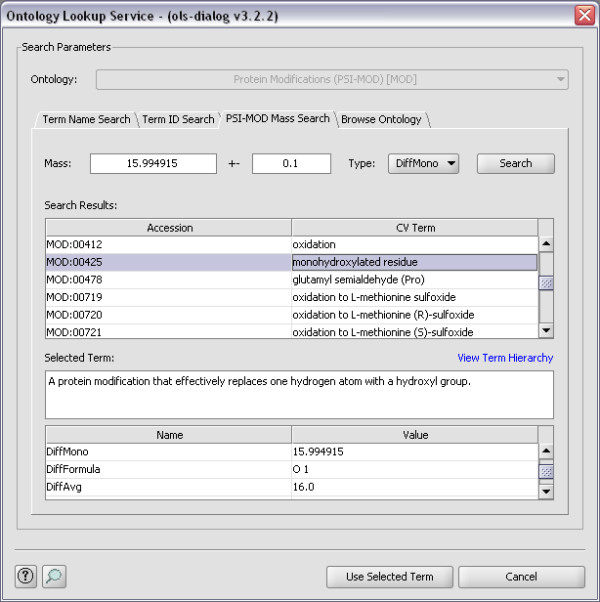
**PSI-MOD Mass Search**. Here the OLS Dialog uses PSI-MOD Mass Search to find the possible CV terms for modifications with a mass of 15.994915 Da with an accuracy of 0.1 Da.

**Figure 3:**
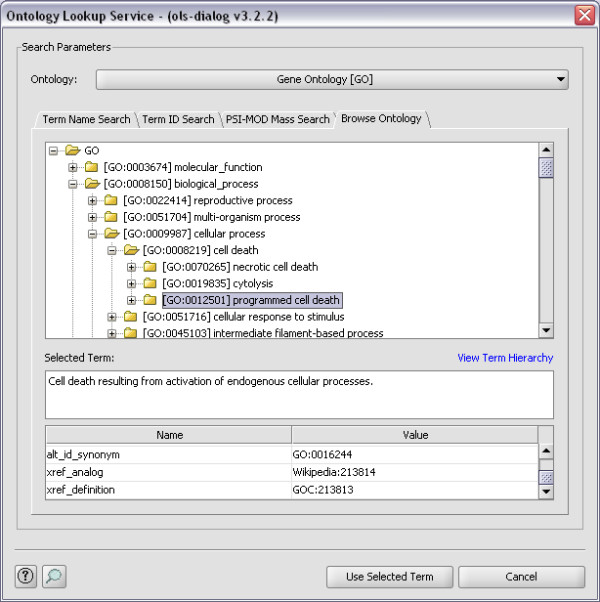
**Browse Ontology**. Here the OLS Dialog is used to locate the term GO:0012501 ('cell death resulting from activation of endogenous cellular processes') in the Gene Ontology using the Browse Ontology feature.

For examples of how the OLS Dialog can be used, see Figure 1, 2 and 3. In Figure 1 Term Name Search is used to find the possible CV terms for the search term 'Oxidation', while in Figure 2 the same term is found using PSI-MOD Mass Search. Figure 3 shows how the Browse Ontology feature can be used to locate the term 'GO:001250'.

To display how the OLS Dialog can be used in other projects we have implemented a simple application, OLS_Example, located in the no.uib.olsdialog.example package. To run the example, download and unzip the OLS Dialog and double click the jar file (or run from the command line using 'java-jar ols-dialog-X.Y.Z', where X.Y.Z represents the version number of the software). More details can be found at the OLS Dialog web page: http://ols-dialog.googlecode.com.

## Conclusions

The OLS Dialog greatly simplifies the usage of the OLS in end-user tools, without requiring any additional knowledge about web services or ontology formats, making it much easier to annotate data using CV terms. The OLS Dialog has already been in use for quite some time in PRIDE Converter [9] for annotating mass spectrometry data. We believe that many other tools could also benefit from the usage of the OLS Dialog, and that this could increase the usage of CV terms for annotating data, which ultimately makes these data even more valuable to the biomedical community.

## Availability and requirements

**Project name**: OLS Dialog

**Project home page**: http://ols-dialog.googlecode.com

**Operating system(s)**: Platform independent

**Programming language**: Java

**Other requirements**: Java 1.5 or newer

**License**: Apache License 2.0 http://www.apache.org/licenses/LICENSE-2.0.html

**Any restrictions to use by non-academics**: none

## List of abbreviations

CV: controlled vocabulary; OLS: Ontology Lookup Service.

## Authors' contributions

HB did the most of part of the programming and contributed in writing the manuscript. RC assisted in the programming and contributed in writing the manuscript. IE monitored the programming and contributed in writing the manuscript. LM monitored the programming and contributed in writing the manuscript. All authors read and approved the final manuscript.
